# Mitochondrial Stress as a Central Player in the Pathogenesis of Hypoxia-Related Myocardial Dysfunction: New Insights

**DOI:** 10.7150/ijms.99359

**Published:** 2024-09-23

**Authors:** Zhijiang Guo, Yingjie Tian, Nanyang Liu, Ye Chen, Xiaohan Chen, Guoxing Yuan, An Chang, Xing Chang, Jie Wu, Hao Zhou

**Affiliations:** 1 Guang'anmen Hospital, China Academy of Chinese Medical Sciences, Beijing, 100053, China.; 2 Beijing University of Chinese Medicine, Beijing, 100028, China.; 3 Xiyuan Hospital, China Academy of Chinese Medical Sciences, Beijing, China.; 4 Senior Department of Cardiology, The Sixth Medical Center of People's Liberation Army General Hospital, Beijing, China.

**Keywords:** Mitochondrial Homeostasis, Hypoxia, Myocardial Injury

## Abstract

Hypoxic injury is a critical pathological factor in the development of various cardiovascular diseases, such as congenital heart disease, myocardial infarction, and heart failure. Mitochondrial quality control is essential for protecting cardiomyocytes from hypoxic damage. Under hypoxic conditions, disruptions in mitochondrial homeostasis result in excessive reactive oxygen species (ROS) production, imbalances in mitochondrial dynamics, and initiate pathological processes including oxidative stress, inflammatory responses, and apoptosis. Targeted interventions to enhance mitochondrial quality control, such as coenzyme Q10 and statins, have shown promise in mitigating hypoxia-induced mitochondrial dysfunction. These treatments offer potential therapeutic strategies for hypoxia-related cardiovascular diseases by regulating mitochondrial fission and fusion, restoring mitochondrial biogenesis, reducing ROS production, and promoting mitophagy.

## 1. Cardiomyocyte injury induced by hypoxia

Myocardial hypoxia refers to abnormal functional alterations in the myocardium caused by insufficient oxygen supply or metabolic disorders and is a leading cause of death in patients with heart disease. When myocardial cells fail to adapt to insufficient oxygen supply, excessive production of reactive oxygen species (ROS) occurs, resulting in mitochondrial quality control dysregulation and inducing apoptosis of myocardial cells 1. Among the clearly defined diseases related to cardiac hypoxia, congenital heart disease (CHD), particularly cyanotic heart defects such as Tetralogy of Fallot and Ebstein's anomaly, is one of the most common heart diseases closely associated with chronic and systemic hypoxemia 2. In addition to myocardial infarction and cardiac hypertrophy, localized hypoxia also accelerates the progression of heart failure 3.

Moreover, hypoxic injury is not only prevalent in various cardiovascular diseases, such as coronary artery disease, heart failure, and myocardial infarction, but also commonly occurs during the treatment of heart-related diseases. For instance, during extracorporeal circulation in cardiac surgery or heart transplantation, the heart experiences hypoxic injury due to reduced or interrupted blood perfusion. After cardiac resuscitation, ischemia-reperfusion (I/R) injury triggers oxidative stress and mitochondrial dysfunction, further exacerbating myocardial damage 4. Additionally, during interventional procedures such as cardiac ablation, reduced blood flow in local tissues may also lead to transient hypoxia, impairing myocardial cells 5. In contrast, the restoration of mitochondrial function has been shown to have markedly beneficial effects on heart failure (HF) caused by myocardial ischemia and hypoxia, such as in ischemia-reperfusion (I/R) injury and myocardial infarction (MI) 6. Therefore, a deeper understanding of the molecular changes in myocardial cells under chronic hypoxic conditions, especially the molecular pathways regulating mitochondrial biogenesis, energy supply, and function, may provide novel therapeutic approaches for diseases associated with cardiac bioenergetic defects, thereby improving myocardial damage and functional failure caused by hypoxia.

### 1.2. Oxidative Stress

The myocardium is a tissue heavily reliant on aerobic metabolism, and mitochondria play a central role in maintaining cardiac function. Under conditions of adequate oxygen supply, the electron transport chain (ETC) in mitochondria efficiently generates ATP through oxidative phosphorylation. However, in a hypoxic environment, the function of the ETC is significantly impaired, particularly at Complex I and Complex III, causing the accumulation of electrons at these sites 6. Under physiological conditions, approximately 1%-2% of electrons leak through Complex I and III to form superoxide anions (O₂⁻). However, under pathological conditions, the production of O₂⁻ exceeds the capacity of the antioxidant system to clear it, leading to a gradual increase in reactive oxygen species (ROS) levels in the mitochondria. Prolonged oxidative stress causes excessive local accumulation of ROS in mitochondria, triggering the mitochondrial permeability transition pore (mPTP) opening, which disrupts the stability of the mitochondrial membrane potential. This change, in turn, further increases ROS production, allowing these reactive molecules to spread to adjacent mitochondria, resulting in a self-propagating chain reaction that exacerbates myocardial cell damage 7, 8.

The excessive generation of ROS under hypoxic conditions is a key factor in myocardial cell damage and dysfunction, primarily through mechanisms involving multiple oxidative damages to lipids, proteins, and DNA. Under hypoxia, ROS-induced lipid oxidation compromises the integrity of the myocardial cell membrane. ROS can induce lipid peroxidation of polyunsaturated fatty acids in the cell membrane, producing lipid peroxides, which decompose into highly bioactive aldehyde products such as malondialdehyde (MDA) and 4-hydroxy-2-nonenal (4-HNE). These products not only compromise membrane integrity but also impair membrane fluidity. Moreover, because ROS generated by mitochondria tend to accumulate locally, mitochondrial lipid membranes are more susceptible to lipid peroxidation than those of other organelles 9.

Additionally, ROS oxidizes the sulfhydryl groups in proteins, promoting the formation of disulfide bonds or inducing protein carbonylation, thereby altering protein conformation and activity. This alteration affects not only intracellular protein metabolism but can also disrupt protein degradation mechanisms, such as the ubiquitin-proteasome system (UPS), ultimately leading to further cellular dysfunction 10. ROS-induced DNA damage primarily manifests as base oxidation and single- or double-strand breaks. If these damages are not repaired in time, they may lead to gene mutations, chromosomal abnormalities, or cell cycle arrest, triggering apoptosis or necrosis 11. Mitochondrial oxidative stress has been found to be closely associated with multiple cardiovascular disease (CVD) risk factors, and both in vitro and in vivo studies have identified it as a major mediator of myocardial cell apoptosis and endothelial dysfunction 12, 13.

### 1.3. Inflammation

Hypoxia-inducible factor 1α (HIF-1α) is a critical regulator of the cellular response to hypoxic stress 14. Under normal oxygen conditions, the protein levels of HIF-1α are regulated by hydroxylases, which hydroxylate HIF-1α, rendering it prone to ubiquitination and rapid degradation. However, under hypoxic conditions, the inhibition of O₂-dependent prolyl hydroxylases-1, -2, and -3 (PHD1, -2, -3) impairs the hydroxylation of HIF-1α. In this state, the HIF-1α protein stabilizes, progressively accumulates in the cytoplasm, and translocates to the nucleus, where it dimerizes with HIF-1β to form a heterodimer complex. This complex binds to hypoxia-responsive elements (HREs) via a common sequence (5'-RCGTG-3') on promoters or enhancers, thereby initiating the transcription of a series of target genes, particularly those involved in inflammatory signaling pathways, promoting the upregulation of pro-inflammatory factors such as IL-6 and TNF-α 15.

Inflammation plays a critical role in cardiovascular diseases, playing a role in processes like atherosclerosis, plaque formation, vascular calcification, and post-ischemic pathological changes, with mitochondrial dysfunction being one of the triggers. In this process, the activation of the mitochondria-mediated NLRP3 inflammasome is considered one of the key mechanisms driving the inflammatory response 16, 17. Under resting conditions, NLRP3 is usually located in the endoplasmic reticulum (ER), but when stimulated by mitochondrial damage-associated molecular patterns (mtDAMPs), NLRP3 aggregates on mitochondria and mitochondria-associated membranes (MAMs). It then recruits apoptosis-associated speck-like protein (ASC; an adaptor) and procaspase-1 (which matures into caspase-1) to modulate the innate immune response and is responsible for the activation of IL-1β and IL-18, triggering an inflammatory response that leads to myocardial cell apoptosis and endothelial dysfunction 18, 19.

### 1.4. Cellular apoptosis

Apoptosis is a controlled process of programmed cell death in multicellular organisms, playing a crucial role in maintaining tissue homeostasis by eliminating damaged or unnecessary cells. The main characteristics of apoptosis include cell shrinkage, chromatin condensation, nuclear fragmentation, membrane blebbing, and the eventual formation of apoptotic bodies 20, 21. Apoptosis can be initiated through two primary pathways: the intrinsic pathway (mitochondrial pathway), regulated by Bcl-2 family proteins that facilitate the release of cytochrome c, and the extrinsic pathway, mediated by the Fas/FasL system. Research has shown that mild hypoxia (1% or 0.1% O₂) induces autophagy to support cell survival without triggering apoptosis. Thus, early hypoxia can induce protective autophagy and moderate apoptosis 22. However, prolonged exposure to hypoxia or extremely low O₂ concentrations (0.1% or 0% O₂) results in widespread cell death 23-25.

In mammalian cells, NIX/BNIP3 and the pro-apoptotic mitochondrial BH3-domain protein BNIP3 play a role in the selective clearance of mitochondria 26, 27. The primary role of BNIP3 and NIX in myocardial cells is to detect myocardial injury and initiate apoptosis through the activation of the pro-apoptotic proteins Bax and Bak 28. Under chronic hypoxic conditions, HIF-1α triggers the mitochondrial apoptosis pathway in myocardial cells by upregulating pro-apoptotic molecules such as BNIP3^29^, ^30^. BNIP3 interacts with Bcl-2 family proteins, increasing mitochondrial membrane permeability, leading to the opening of the mitochondrial permeability transition pore, disrupting mitochondrial membrane potential, and accelerating the release of cytochrome c (Cyt c) and apoptosis-inducing factor (AIF)^31^, which activates downstream caspase cascades and exacerbates myocardial cell apoptosis^32^, ^33^. Furthermore, hypoxia disrupts calcium homeostasis in myocardial cells, leading to calcium overload, which exacerbates mitochondrial damage and triggers endoplasmic reticulum stress, further activating pro-apoptotic molecules such as CHOP and caspase-12 31, 34.

Under hypoxic conditions, BNIP3 expression is significantly upregulated in the myocardium, while NIX is primarily upregulated in pathological cardiac hypertrophy 35-37. Excessive apoptosis of myocardial cells not only aggravates myocardial damage but also promotes a range of pathological changes, such as cardiac remodeling and coronary atherosclerosis, ultimately leading to severe consequences such as heart failure 38-40.

## 2. Mitophagy and Hypoxia-Induced Programmed Cardiomyocyte Death

Mitophagy refers to the selective clearance of mitochondria via autophagic processes, involving multiple biological stages such as the recognition of damaged mitochondria, their encapsulation, autophagosome formation, lysosomal fusion, and degradation 41, 42. Mitophagy is primarily mediated by the PINK1/Parkin signaling pathway, where the loss of mitochondrial membrane potential serves as a key signal to initiate the process. When mitochondrial membrane potential decreases, PINK1 localizes to the outer mitochondrial membrane, recruiting and activating the E3 ubiquitin ligase Parkin 25, 43. Parkin conjugates ubiquitin to proteins on the outer mitochondrial membrane, marking these mitochondria for recognition and encapsulation by autophagosomes. Autophagosomes subsequently fuse with lysosomes to form autolysosomes, degrading mitochondria into essential components such as amino acids and lipids for cellular reuse. Mitophagy preserves cellular viability by degrading damaged proteins. Research on myocardial ischemia-reperfusion injury (MIRI) has shown that mitophagy removes damaged mitochondria, sustaining intracellular homeostasis and myocardial cell function 44.

Hypoxia typically activates mitophagy, with HIF-1α serving as a crucial regulatory factor in this process 45. Under hypoxic conditions, the degradation of HIF-1α is inhibited, and upon stabilization, it translocates to the nucleus, where it binds to hypoxia-responsive elements (HREs) in target genes, modulating the expression of essential proteins related to mitophagy. Among them, BNIP3 acts as a core regulator of mitophagy by interacting with LC3 to facilitate the selective clearance of damaged mitochondria 46, helping to maintain mitochondrial homeostasis and reducing oxidative stress. Studies have demonstrated that ischemia-reperfusion (I/R) injury significantly increases HIF-1α expression, which in turn activates downstream BNIP3, triggering mitochondria-dependent autophagy to counteract excessive ROS production and energy metabolism imbalance during I/R 40. This protective mechanism is vital for myocardial cell survival, especially under hypoxic or ischemic conditions, as mitophagy mitigates apoptosis through the removal of damaged mitochondria 47. The HIF-1α/BNIP3 pathway provides a new direction for developing targeted therapies for hypoxia-related myocardial injury. Further research has shown that berberine (BBR) effectively induces mitophagy by activating this pathway, clearing dysfunctional mitochondria, inhibiting myocardial apoptosis, promoting cell proliferation and repair, and alleviating myocardial damage caused by I/R injury 45.

However, under severe hypoxia, mitochondrial dysfunction and oxidative stress may inhibit the normal progression of mitophagy. Excessive ROS not only cause mitochondrial damage but may also oxidize and damage key mitophagy-related proteins, hindering the autophagic process. Additionally, other cellular stress responses, such as the unfolded protein response (UPR), and programmed cell death pathways like apoptosis, necroptosis, and pyroptosis, may compete for cellular energy and resources, further reducing the efficiency of mitophagy 48. This competition for resources is particularly prominent under severe hypoxia, potentially hindering mitophagy's protective effects and exacerbating cell damage and myocardial dysfunction 49, 50.

### 2.1. Mitochondrial Dynamics and Hypoxia-Induced Programmed Cardiomyocyte Death

Mitochondria are often regarded as independent organelles, yet in reality, they form a highly dynamic network. Their structure and distribution profoundly influence metabolic function, with the properties of these complex networks depending on the balance between mitochondrial fission and fusion. Mitochondrial fusion is a key process in mitochondrial dynamics, integrating and optimizing mitochondrial function through the merging of multiple mitochondria into a larger structure 48. This process begins at the outer mitochondrial membrane and is mediated by the fusion proteins Mitofusin 1 (Mfn1) and Mitofusin 2 (Mfn2), which are found in the outer mitochondrial membrane. Mfn1 and Mfn2 recognize and bind to each other on adjacent mitochondria, forming homotypic or heterotypic complexes that promote outer membrane fusion. After this, inner membrane fusion is mediated by the Opa1 (Optic atrophy 1) protein, which collaborates with pro-fusion proteins such as Yme1L and PARL to facilitate inner membrane fusion.

Mitochondrial fusion promotes the mixing of DNA, proteins, lipids, and other metabolites within the mitochondrial matrix across different mitochondria, ensuring even distribution of the mitochondrial genome (mtDNA) and preserving mitochondrial genetic integrity. Fusion also allows damaged mitochondria to interact with their healthy counterparts, facilitating content exchange that repairs or dilutes damaged components and enhances overall mitochondrial function 51. Moreover, fusion increases the volume and complexity of the mitochondrial network, enabling more efficient ATP production to fulfill cellular energy demands under various physiological conditions. In contrast, mitochondrial fission refers to the process by which a single mitochondrion divides into two or more smaller mitochondria. The outer membrane fission is primarily mediated by the cytosolic protein Drp1 (Dynamin-related protein 1) 51. Fission not only facilitates the removal of damaged mitochondria through mitophagy but also increases mitochondrial surface area, boosting ATP generation to meet increased cellular energy demands. In cardiomyocytes, the balance of mitochondrial fusion and fission is critical, dynamically regulating the mitochondrial network to maintain cellular energy homeostasis and ensuring that the heart receives the high energy supply necessary for continuous contraction 50.

Hypoxia generally induces mitochondrial fission 52. Under low-oxygen conditions, DRP1 expression is upregulated and activated, recruiting to the outer mitochondrial membrane and promoting mitochondrial fission, resulting in mitochondrial fragmentation and separation of damaged mitochondrial fragments. These fragmented mitochondria are more susceptible to damage and become sources of excessive reactive oxygen species (ROS), exacerbating mitochondrial dysfunction 53. The effects of hypoxia on mitochondrial fusion, however, are more complex. Studies have shown that under controlled hypoxic conditions, cells may temporarily enhance mitochondrial fusion to dilute damaged mitochondrial components and prevent excessive mitochondrial clearance. However, under severe oxidative stress, hypoxia impairs the expression and activity of fusion proteins like OPA1, impairing mitochondrial fusion and further aggravating mitochondrial dysfunction. Mouse model studies have demonstrated that stress-induced OMA1 activation accelerates OPA1 cleavage, leading to excessive mitochondrial fragmentation in cardiomyocytes and neurons, ultimately triggering cell death. Conversely, deleting OMA1 or inhibiting excessive mitochondrial fission (e.g., through DRP1 inhibition) has been shown to significantly protect cardiomyocytes 54.

### 2.2. Mitochondrial Biogenesis and Hypoxia-Induced Programmed Cardiomyocyte Death

Mitochondrial biogenesis refers to the process of increasing the number of mitochondria, optimizing their function, and refining their structural integrity through the precise regulation of mitochondrial DNA (mtDNA) replication, transcription and translation of mitochondrial proteins, and the assembly of mitochondrial membranes and functional components. Key steps in this process include the transcription of nuclear-encoded genes, replication of mtDNA, synthesis and import of mitochondrial proteins, and the assembly of components such as mitochondrial membranes and respiratory chain complexes. Mitochondrial biogenesis is a balanced process that occurs in tandem with mitophagy to maintain the optimal mitochondrial population within cells. Pretide is a mitochondria-targeted peptide drug that binds directly to cardiolipin in the mitochondrial inner membrane. By stabilizing the mitochondrial inner membrane structure, it inhibits oxidative stress triggered by excessive reactive oxygen species (ROS) production, thereby preventing cardiomyocyte damage during hypoxia or oxidative stress. Long-term treatment with Elamipretide has been shown to improve left ventricular systolic function in canines with advanced heart failure. Elamipretide not only normalizes plasma levels of tumor necrosis factor-α (TNF-α) and C-reactive protein (CRP) but also significantly improves mitochondrial function, including the restoration of state-3 respiration, mitochondrial membrane potential (Δψm), ATP synthesis rate, and ATP/ADP ratio.Studies have also found that in both dogs and humans with heart failure (HF), mitochondrial dynamics in the left ventricular myocardium are impaired, with disrupted mitochondrial biogenesis, imbalances in fission and fusion mechanisms, and downregulation of mitofusins. Elamipretide selectively targets mitochondria and reverses these abnormalities, restoring mitochondrial dynamics, thus demonstrating significant potential to enhance myocardial function and counteract heart failure 55.

## 3. Targeted Pharmacological Therapy

### 3.1. Coenzyme Q10

Coenzyme Q10 (CoQ10) is an organic molecule widely present in cell membranes, particularly within mitochondria, in both its reduced form (ubiquinol) and oxidized form (ubiquinone). Several mitochondrial proteins (MXPs) that are not fully characterized are closely involved in the biosynthesis of CoQ10. Clinical studies have shown that CoQ10 has potential therapeutic effects in cardiovascular diseases, and exogenous CoQ10 supplements can serve as adjunctive therapy for heart failure, atrial fibrillation, myocardial infarction, and other cardiovascular diseases and their associated risk factors, such as hypertension, insulin resistance, dyslipidemia, and obesity. In randomized controlled clinical trials (RCTs), patients taking CoQ10 demonstrated significant reductions in markers of inflammation and oxidative stress, providing further evidence for its potential in the treatment of cardiovascular diseases 56. Mitochondria-targeted nanocarriers deliver CoQ10 to the mitochondria of cardiomyocytes subjected to cold ischemia-reperfusion injury. Studies have shown that this delivery method effectively scavenges mitochondrial reactive oxygen species (mtROS), reducing oxidative damage and inflammation in the transplanted tissue, ultimately improving the function of the transplanted heart 57.

### 3.2. Statins

Statins are a class of drugs commonly used to treat hyperlipidemia and are widely utilized in clinical practice due to their significant role in preventing cardiovascular events. Statins reduce endogenous cholesterol synthesis by inhibiting β-hydroxy-β-methylglutaryl-coenzyme A (HMG-CoA) reductase, the rate-limiting enzyme in the mevalonate pathway. In addition to lowering cholesterol, statins also provide various "pleiotropic effects" beneficial to the cardiovascular system, such as improving endothelial function, providing antioxidant and anti-inflammatory benefits, and stabilizing atherosclerotic plaques. The cardioprotective effects of statins partially depend on the activation of mitophagy, a mechanism particularly important in mitigating hypoxic injury in cardiomyocytes. Studies have shown that in a mouse model of myocardial infarction, simvastatin significantly reduced infarct size in wild-type mice, but this protection was lost in Parkin gene knockout mice 58. Furthermore, studies have shown that in the hearts of patients treated with statins, reactive oxygen species (ROS) production decreases, antioxidant capacity increases, and the mRNA expression of the peroxisome proliferator-activated receptor gamma coactivator (PGC-1) family is significantly upregulated, further suggesting that statins promote mitochondrial biogenesis in the heart and enhance antioxidant capacity 59.

### 3.3. Erythropoietin (SS-31)

Elamipretide is a mitochondria-targeted peptide drug that binds directly to cardiolipin in the mitochondrial inner membrane. By stabilizing the mitochondrial inner membrane structure, it inhibits oxidative stress triggered by excessive reactive oxygen species (ROS) production, thereby preventing cardiomyocyte damage during hypoxia or oxidative stress. Long-term treatment with Elamipretide has been shown to improve left ventricular systolic function in canines with advanced heart failure. Elamipretide not only normalizes plasma levels of tumor necrosis factor-α (TNF-α) and C-reactive protein (CRP) but also significantly improves mitochondrial function, including the restoration of state-3 respiration, mitochondrial membrane potential (Δψm), ATP synthesis rate, and ATP/ADP ratio.Studies have also found that in both dogs and humans with heart failure (HF), mitochondrial dynamics in the left ventricular myocardium are impaired, with disrupted mitochondrial biogenesis, imbalances in fission and fusion mechanisms, and downregulation of mitofusins. Elamipretide selectively targets mitochondria and reverses these abnormalities, restoring mitochondrial dynamics, thus demonstrating significant potential to enhance myocardial function and counteract heart failure 55.

### 3.4. Metformin

Metformin, a drug commonly used to treat type 2 diabetes, has recently been found to have significant cardioprotective effects. In a rat model of myocardial ischemia-reperfusion injury (MIRI), studies have shown that metformin significantly reduces infarct size and lowers plasma levels of lactate dehydrogenase and creatine kinase-MB. The mechanism underlying its cardioprotective effects is related to the activation of the AMPK pathway, which inhibits NOX4 expression. Downregulation of NOX4 reduces myocardial oxidative damage and apoptosis, thereby effectively mitigating reperfusion-induced myocardial injury 60.

However, metformin has also been shown to exhibit adverse effects on therapeutic outcomes in certain cases. Research indicates that metformin may reduce the therapeutic efficacy of mesenchymal stem cells (MSCs), increase infarct size, and limit cardiac functional recovery. Specifically, metformin induces AMPK-mediated apoptosis by inhibiting the S6K1-Bad-Bcl-xL cell survival signaling pathway, leading to upregulation of apoptosis-related proteins and increased MSC apoptosis 61. In addition, metformin has shown potential in reducing hypoxia-induced myocardial inflammation in inflammation-related studies. This mechanism is related to the regulation of Th17 cell-mediated inflammatory responses through increased autophagy and improved mitochondrial bioenergetics 62.

## Figures and Tables

**Figure 1:**
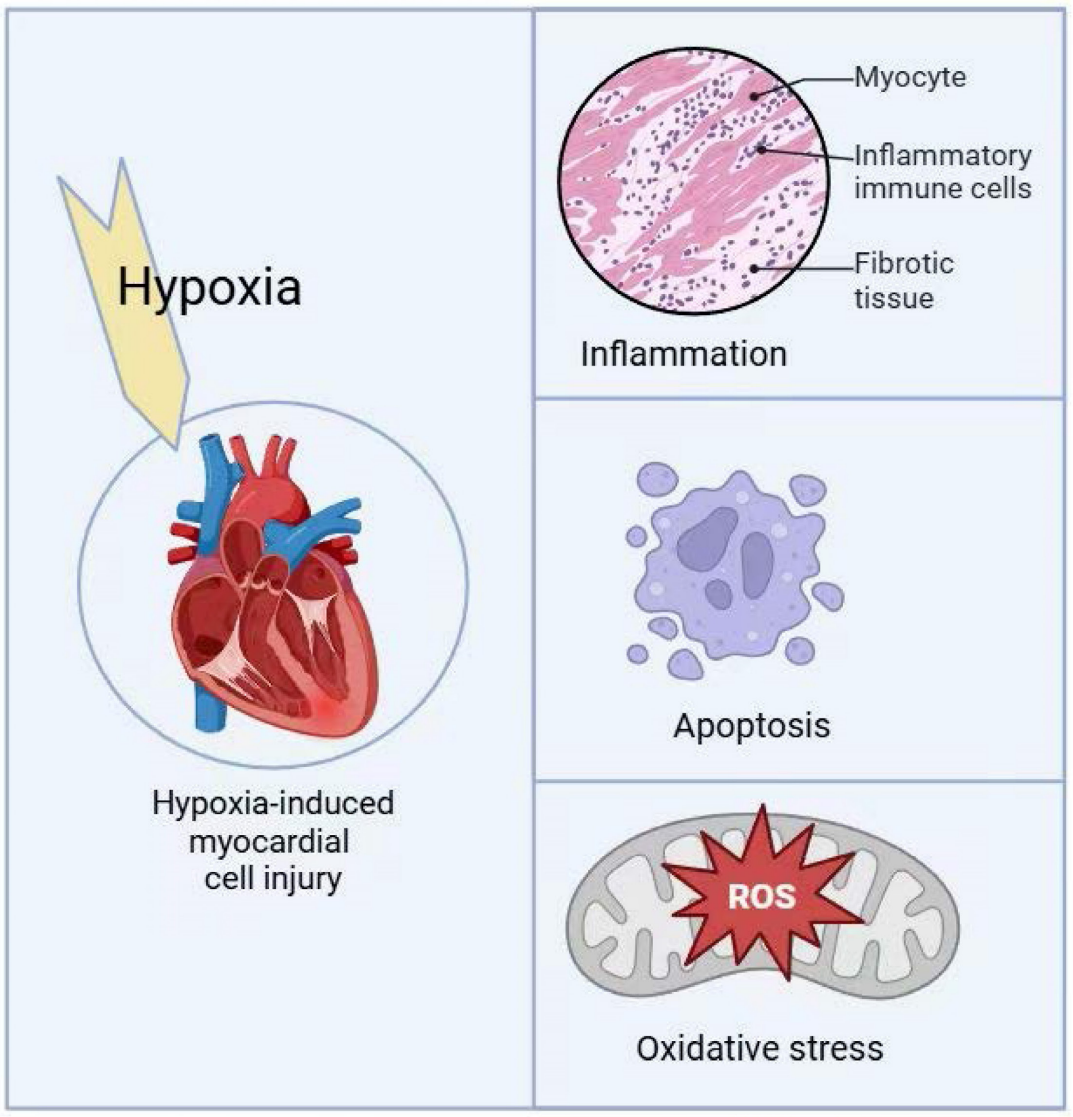
Hypoxia-induced myocardial cell injury mechanisms.

**Table 1:** Targeted Pharmacological Therapy for Hypoxia-related Myocardial Injury

Name	Target	Reference
Coenzyme Q10	mitochondrial function↑; mitophagy↓; ROS↓; Inflammation↓	63, 64, 57, 65, 66
Statins	mitophagy↑; ROS↓, PGC-1 mRNA↓, mitochondrial biogenesis↑	58, 59
Elamipretide	mitochondrial function↑; mitochondrial dynamics↑; ROS↓	67, 68
Metformin	mitochondrial function↑; ROS↓; Apoptosis↓; mitochondrial dynamics↑;	69, 70
